# Supplementary material for: Cystic Fibrosis Cases Missed by Newborn Bloodspot Screening—Towards a Consistent Definition and Data Acquisition
Source: Int J Neonatal Screen. 2023 Nov 21;9(4):65. doi: 10.3390/ijns9040065 (PMC10743499; doi:10.3390/ijns9040065)
Supplement: Supplementary file 1 [file IJNS-09-00065-s001.zip › IJNS-2718686-supplementary.pdf]

Current strategies for collecting missed cases*

Only one reply by region or country

Filled in by ………………………………………………………………… Position ……………………………………………

Date ………… /…………… /………………

Country/Region: …………………………………………………….……………………

☐ National CF-NBS programme ☐ Regional CF-NBS programme

---

1. For how many years have you collected data on missed cases in your country/region?

☐ 2 years ☐ 3 years ☐ 4 years ☐ 5-9 years ☐ ≥ 10 years

2. Strategies to identify missed cases in <u>2023</u> (*More than one answer is possible)*

- ☐ Annual questionnaire by the national/regional coordinator or structure
  - ☐ sent to the head of paediatric CF centres
  - ☐ sent to the regional screening centre
  - ☐ sent to the screening laboratory
- ☐ Paediatric CF centre fills in an assessment sheet when a child is clinically diagnosed having CF
  - ☐ sent to the regional/national coordinator
  - ☐ sent to the regional/national screening laboratory
- ☐ Regular meetings for reporting missed cases (at least annually) between CF physicians and the national/regional coordinator
- ☐ CF registry (national or European)
- ☐ Unknown strategy

☐ Other: ……..

3. Which structure is in place to ensure collection of completed questionnaires (incl. reminders)?

- ☐ Centralized coordinator or structure at national level
- ☐ Centralized coordinator or structure at regional level
- ☐ No structure (no centralised coordination)
- ☐ Unknown
- ☐ Other: ………………………………………………………………………………………………………..

---

* defined as all missed cases, consisting

1. NBS protocol related (→ analytical issues)
2. Not NBS protocol related (→ pre- and post-analytical issues)

4. Did the strategy collecting missed cases change in the last few years (since the EU survey for 2019 data)?

- ☐ no
- ☐ yes: How did this change? ……..……………………………………………………………………
- ☐ Unknown

5. Are the missed cases reported in your programme?

- ☐ no
- ☐ yes

6. If they are reported, are the missed cases classified based on the criteria below?

a) Diagnosed based on symptoms	☐ yes	☐ no	☐ unknown
b) Meconium ileus (with normal IRT)	☐ yes	☐ no	☐ unknown
c) Antenatal diagnosis (with normal IRT)	☐ yes	☐ no	☐ unknown
d) Exposure to CFTR modulator during pregnancy	☐ yes	☐ no	☐ unknown

7. How many years after birth maybe needed to identify the majority (≥80%) of missed cases in your country/region?

☐ 2 years ☐ 3 years ☐ 4 years ☐ 5-9 years ☐ ≥ 10 years

8. What percentage of missed cases is currently collected in 2023 in your country/region?

☐ ≥80% ☐ 60-79% ☐ 40-59% ☐ ≤40% ☐ no idea

If <80% what are the reasons for missing data in your region/country:

……………………………………………………………………………………………………….

9. Are you able to collect these items at diagnosis for all identified missed cases?

• <u>Date of birth</u> (month/year)	☐ yes	☐ no	☐ unknown	☐ not permitted to collect
• <u>Date of CF diagnosis</u> (month/year)	☐ yes	☐ no	☐ unknown	☐ not permitted to collect
• <u>IRT-1 / PAP-values:</u>	☐ yes	☐ no	☐ unknown	☐ not permitted to collect
• <u>IRT-2 / safety net:</u>	☐ yes	☐ no	☐ unknown	☐ not permitted to collect
• <u>Sweat test result:</u>	☐ yes	☐ no	☐ unknown	☐ not permitted to collect
• <u>Genotype (CFTR mutation):</u>	☐ yes	☐ no	☐ unknown	☐ not permitted to collect
• <u>Exocrine pancreatic status:</u>	☐ yes	☐ no	☐ unknown	☐ not permitted to collect

- Symptoms & family history (*More than one answer is possible)*

	YES	NO	unknown
1. Familial CF case			
2. Respiratory symptoms			
3. Gastrointestinal symptoms			
4. Failure to thrive			
5. Rectal prolapse			
6. Dehydration			
7. Liver disease			
8. Nasal polyps			
9. Other: provide the list with all other recorded symptoms			
Unknown circumstances			

- Sample collection and/or Laboratory problems

	YES	NO	unknown
**Sample collection**			
1. Dry blood spot (DBS) sample is not obtained or lost			
2. DBS sample labelling error in the neonatal nursery			
3. DBS sample mix-up			
4. DBS sample quality is unacceptable			
5. DBS taken on wrong day (too early/too late)			
6. Mother taken Elexacaftor-Tezacaftor-Ivacaftor (ETI) during pregnancy (not reported)			
**In the laboratory**			
5. First IRT (IRT-1) or IRT-1/PAP values above cut offs are not actioned			
6. Infant's IRT-1 or IRT-1/PAP level is below the cut off			
7. In IRT/IRT protocol: A 2nd specimen is not obtained, no follow-up			
8. In IRT/IRT protocol: The second IRT (IRT-2) is below the cut off			
9. In IRT/DNA protocol: Variants not in the panel			
10. In IRT/DNA protocol: Failure in DNA analysis			
11. In IRT/DNA protocol: No detected variant and initial IRT-1 is below the ultrahigh cut-off for next step IRT-2			

12. In IRT/DNA protocol: no detected variant and ultrahigh IRT-1, next step IRT-2 is below the cut-off			
13. Administrative error in reporting the NBS result to the CF team/primary care provider/family			

- Communication problems & diagnostic issues

**Follow-up**	YES	NO	unknown
14. Miscommunication of NBS result between primary care provider and family (e.g., sweat test not performed, family not reachable, not responding or not willing to come)			
15. Error in measurement of sweat chloride concentration (SCC)			
16. Appropriate cut-off values of SCC are not used			
17. SCC is normal after detection of only one variant (in IRT/DNA protocol)			

10. Would you agree to complete a more detailed questionnaire for all missed cases born in 2019 for your region/country?

☐ yes ☐ no if no, why: ………………………………………………….

11. What are the main challenges to collecting data on missed cases in your programme?

☐ ……………………………………………………………………………………………………….
